# Evaluation of guided imagery as treatment for recurrent abdominal pain in children: a randomized controlled trial

**DOI:** 10.1186/1471-2431-6-29

**Published:** 2006-11-08

**Authors:** Joy A Weydert, Daniel E Shapiro, Sari A Acra, Cynthia J Monheim, Andrea S Chambers, Thomas M Ball

**Affiliations:** 1 Integrative Pain Management, Children's Mercy Hospital, 2401 Gillham Road, Kansas City, Missouri 64108 USA; 2 Department of Pediatrics, University of Arizona Health Sciences Center, 1501 N. Campbell Avenue, Tucson, Arizona 85724 USA; 3 Steele Memorial Children's Research Center, University of Arizona Health Sciences Center, 1501 N. Campbell Avenue, Tucson, Arizona 85724 USA; 4 Program in Integrative Medicine, University of Arizona Health Sciences Center, 1501 N. Campbell Avenue, Tucson, Arizona 85724 USA; 5 Department of Psychiatry, University of Arizona Health Sciences Center, 1501 N. Campbell Avenue, Tucson, Arizona 85724 USA; 6 Department of Psychology, University of Arizona Health Sciences Center, 1501 N. Campbell Avenue, Tucson, Arizona 85724 USA; 7 Department of Pediatrics, Vanderbilt University Medical Center, S-4322 MCN 2578, 3601 The Vanderbilt Clinic, Nashville, Tennessee 37232 USA

## Abstract

**Background:**

Because of the paucity of effective evidence-based therapies for children with recurrent abdominal pain, we evaluated the therapeutic effect of guided imagery, a well-studied self-regulation technique.

**Methods:**

22 children, aged 5 – 18 years, were randomized to learn either breathing exercises alone or guided imagery with progressive muscle relaxation. Both groups had 4-weekly sessions with a therapist. Children reported the numbers of days with pain, the pain intensity, and missed activities due to abdominal pain using a daily pain diary collected at baseline and during the intervention. Monthly phone calls to the children reported the number of days with pain and the number of days of missed activities experienced during the month of and month following the intervention. Children with ≤ 4 days of pain/month and no missed activities due to pain were defined as being healed. Depression, anxiety, and somatization were measured in both children and parents at baseline.

**Results:**

At baseline the children who received guided imagery had more days of pain during the preceding month (23 vs. 14 days, P = 0.04). There were no differences in the intensity of painful episodes or any baseline psychological factors between the two groups. Children who learned guided imagery with progressive muscle relaxation had significantly greater decrease in the number of days with pain than those learning breathing exercises alone after one (67% vs. 21%, P = 0.05), and two (82% vs. 45%, P < 0.01) months and significantly greater decrease in days with missed activities at one (85% vs. 15%, P = 0.02) and two (95% vs. 77%. P = 0.05) months. During the two months of follow-up, more children who had learned guided imagery met the threshold of ≤ 4 day of pain each month and no missed activities (RR = 7.3, 95%CI [1.1,48.6]) than children who learned only the breathing exercises.

**Conclusion:**

The therapeutic efficacy of guided imagery with progressive muscle relaxation found in this study is consistent with our present understanding of the pathophysiology of recurrent abdominal pain in children. Although unfamiliar to many pediatricians, guided imagery is a simple, noninvasive therapy with potential benefit for treating children with RAP.

## Background

Chronic pain is a significant problem in the pediatric population [1]. One of the more common chronic pain syndromes in children is recurrent abdominal pain (RAP) thought to affect 10–30% of all school-aged children [2-5]. RAP is characterized by the recurrence of a minimum of three episodes of abdominal pain within a 3-month period severe enough to hinder the child's activities [2]. Children with RAP were found to miss 21 more days of school per year[6] and have higher levels of anxiety and depression than age-matched controls [7,8]. Many of these children go on to become adults with chronic abdominal pain or anxiety disorders [9]. Therefore, an ideal therapy for childhood RAP, considered a functional gastrointestinal disorder, would not only decrease pain in the short term, but also potentially improve functioning in the long term.

Recent systematic reviews of the treatments for functional gastrointestinal disorders in children found weak evidence for the effectiveness of very few therapies [10-12]. Two pharmaceuticals and one botanical therapy were found to have some efficacy in specific subtypes of functional abdominal pain [13-15]. No dietary manipulations were found to be efficacious [16-19]. Although cognitive-behavioral approaches were found to work well for children with non-specific RAP, they are infrequently offered to patients [20-23].

In attempts to understand the nature of functional gastrointestinal disorders researchers have focused on the functioning of the enteric nervous system (ENS). The ENS acts as a local minibrain producing the same neuropeptides and neurotransmitters found in the central nervous system (CNS) [24]. These act locally to regulate gastrointestinal motility, blood flow, secretions, and absorption [25]. The CNS, in turn, has its own effects on the ENS. Stress is known to aggravate the gastrointestinal tract through the release of neuropeptides and neurotransmitters triggering various gastrointestinal responses.

Because of these recent findings, our current understanding of functional gastrointestinal disorders has moved from a biomechanical model towards one with a biopsychosocial emphasis [26]. This understanding has led to the increasing use of self-regulation therapies for treating such pain syndromes. These therapies often referred to as mind-body therapies include hypnosis, biofeedback, guided imagery, meditation, and relaxation techniques. They are theorized to work through action on neurotransmitters and catecholamines that influence the mind's perception of pain thereby decreasing sympathetic drive [27]. Documented physiologic responses to relaxation include decreased oxygen consumption, blood pressure, heart rate, serum lactic acid levels, and tonic muscle tension [28]. One small case-series specifically evaluated the use of hypnosis in children with RAP and found that 4 out of 5 children improved with this intervention [29]. A larger study completed in adults with irritable bowel syndrome evaluated the long-term outcomes of hypnosis [30]. They found the beneficial effects lasted at least 5 years in 71% of their patients who completed the therapy.

Guided imagery is one form of self-regulation therapy. During the process, a state of deep relaxation is induced using progressive muscle relaxation (PMR) which allows the subject to then be guided in actively creating images that facilitate resolution of certain problems. This differs from hypnosis in that the child, through imagery, creates his own solution to the problem rather than the therapist offering suggestions for change. This process is felt to be especially effective in children because of their ability to have creative, active imaginations with a high degree of suggestibility [31]. We consciously use words and logic in the process of thinking which is primarily the function of the 'left brain'. However, it is our 'right brain' that processes information more in terms of images, feelings, and emotions at the unconscious level [32]. A recent study of 59 children with RAP found that they tended to have greater subliminal attentional biases toward pain-related words [33]. The use of guided imagery allows for communication with that subliminal part of the mind to create change. Communication through images, along with the deep relaxation, reduces anxiety which frequently has components of voluntary and autonomic nervous system hyper-reactivity which contributes to pain [34,35]. In one study of children with RAP, all were treated with guided imagery and progressive muscle relaxation techniques. There was no control group. Follow-up done over the next 10 months revealed that 89% had improvement of their pain. Additionally, they had less missed days of school and increased activity levels [36].

This makes guided imagery a potentially ideal modality for treating children with functional gastrointestinal disorders. With physical relaxation and behavior modification through imagery, there may be regulation of gastrointestinal motility and an increase in the visceral pain threshold in these children. Because of our success with this technique during a pilot study [37], we conducted this randomized and controlled study to examine the efficacy of guided imagery compared to breathing exercises alone for the treatment of recurrent abdominal pain in children.

## Methods

The methodology of this study was based on the guidelines outlined by an international panel of experts that convened for the design, conduct, and analysis of treatments trials in functional gastrointestinal disorders (Rome II) [38]. Additionally, guidelines proposed by the CONSORT statement were followed to improve the reporting of this randomized, controlled trial and minimize systematic error [39].

### Study population

Children 5–18 years of age were recruited from pediatric gastroenterologists within the University of Arizona Department of Pediatrics and general pediatricians throughout the greater Tucson metropolitan area. Inclusion criteria included a history of at least 3 episodes of abdominal pain over the previous 3 months severe enough to affect their normal activity. All participants had a complete history and physical performed by their pediatrician or pediatric gastroenterologist and had a minimal laboratory evaluation including a complete blood count, sedimentation rate, and urinalysis. All other diagnostic tests were performed at the discretion of the treating physician. Participants were required to be stable on any current medications they were taking and asked not to add, delete, or change the dosing of any medication for the duration of the study. Participants were required to be English-speaking. Exclusion criteria were unwillingness to participate, chronic documented gastrointestinal disease, cognitive-developmental delay or major dissociative disorder as determined by history as the latter two are contraindications for doing guided imagery. The Institutional Review Board of the University of Arizona approved this study. Informed consent and assent for participation were obtained from the parents and children, respectively.

### Measurement of abdominal pain and disability

Daily pain diaries were obtained at baseline by the child for at least 2 weeks prior to the start of the intervention and during the first month of the intervention. These were used to record the number of days with pain and to rate the intensity of pain experienced. To rate the intensity children used the FACES scale [40] of 0–6 for any pain noted at 7 AM, 2 PM, and 6 PM each day. In addition, children, along with their parents, documented any days the child missed a normal activity (i.e. school, sports, social activities, etc.) because of abdominal pain. Because we found compliance with diary completion worsened by the end of the first month of the intervention during our pilot study, we also called each family at 1 and 2 months to ascertain the estimated number of days of pain and number of days with missed activities during the previous month. Intensity measures of pain episodes were not collected by telephone and therefore are not available after the first month of intervention. These telephone reports correlated highly with the children's daily pain diary reports and are therefore used here [37,41].

### Symptom and Psychological factors

Baseline questionnaires to obtain symptom and psychological measures were completed both by the child and the parent. These included, for the child, the Bowel Symptom Questionnaire (BSQ) adapted from Talley's instrument for use in children [42], Child Depression Inventory (CDI) [43], Multidimensional Anxiety Scale for Children (MASC) [44], EAS Temperament Scale [45], and Child Somatization Inventory (CSI) [46]. Because reviews of previous studies demonstrated differences between children with RAP and normal controls without abdominal pain [7,8,47] measures of anxiety, depression, and somatization were included in this analysis. In the parents, the Symptom Checklist-90 (SCL-90) and the Parent Bonding Instrument (PBI) were used to assess anxiety, depression, somatization, and parenting styles [48,49].

### Breathing and Guided Imagery therapy

Our hypothesis was that guided imagery with progressive muscle relaxation would be superior to breathing techniques alone for managing the symptoms of functional gastrointestinal disorders, therefore subjects were randomized to receive either breathing techniques alone or guided imagery with PMR. Two randomized tables were used depending on the source of the referral–pediatric gastroenterologist or general pediatrician–and further stratified by age, grouped age 5 < 12 and = 12 to 18. Random assignment was made in groups of four by drawing tokens out of a hat by the biostatistics core group assisting with this study. This randomization list was given only to the therapists teaching the breathing techniques and guided imagery and was concealed until the intervention was assigned. No other member of the research team was aware of the group assignments. All treatments, regardless of the group, were referred to as "relaxation techniques", which allowed blinding of the research associate collecting outcomes and some degree of masking of subjects not previously aware of these therapies.

For those randomized to receive guided imagery, four sessions were done on a weekly basis. During the first session, which lasted about 1 hour, participants were instructed on progressive muscle relaxation which led into the guided imagery. Once achieving relaxation, subjects were asked to invite an image to come to mind that represented their pain. They were encouraged to describe the image in detail using all the senses as the more detailed the image is sensed, the more potential the pain reliever it could be. Once this image was established, they were then asked to invite a second image to come that would get rid of the pain (first image). An audiotape of the relaxation and imagery was sent with the subject to practice at home twice daily. Three follow up sessions, which lasted 20–30 minutes, were done to assess competence, to assess compliance with daily practice, and for reinstruction and reinforcement. No psychotherapy or further counseling occurred during these sessions.

The control group was designed to mimic the intervention in order to control for the therapist's time and attention. Those randomized to receive the breathing techniques also met weekly with the therapist for 4 sessions. During the first session of approximately 1 hour, subjects were instructed on 3 breathing techniques that facilitate relaxation: abdominal breathing, "breathing in fives" (inhale fully and deeply for a count of 5, hold for 5, fully exhale for a count of 5, and hold for 5), and "bubble breathing" (slow sustained exhalation using soap bubbles and a wand to create 1 large bubble or a steady stream of smaller bubbles). After practicing these techniques to achieve relaxation, an audiotape was made for subjects to take with them to practice at home twice daily, repeating each of the three exercises 5 times apiece. At each of the 4 sessions, subjects were evaluated for their competence in performing these exercises to achieve relaxation. In the 3 follow up sessions that lasted approximately 20–30 minutes, assessment of compliance of practice, and if needed, reinstruction of the techniques was performed.

### Statistical Analysis

Pain outcomes measured were number of days with pain per month, mean pain intensity per pain episode, and the number of days with a missed activity due to abdominal pain per month. However, since the Rome II expert panel recommended that the primary outcome measure be the percentage of subjects meeting a predetermined clinical outcome, we used as our primary outcome the percentage of children who had ≤ 4 days with pain and no missed activities during the previous month [41]. The term "healed" was chosen as the term for our primary outcome to reflect the fact that RAP may be a relapsing disorder and not necessarily curable but implies restored functionality and quality of daily living. In order to assess the longitudinal response to treatment the generalized estimation equation was used to analyze the probability of being healed at months 1 and 2. This is a longitudinal statistical procedure that provides estimates for mixed-effects regression models for longitudinal dichotomous data [50]. Children were required to have complete information at baseline and months 1 and 2 in order to be included in the analysis of being healed. All factors associated with the treatment group and being healed with a P < 0.10 were considered as potential confounders and evaluated for their impact on the adjusted relative risks.

All analyses were compared using Chi-square or Student's t-test after transformation of non-normally distributed continuous variables when appropriate. An alpha of < 0.05, two-sided, was considered statistically significant. Because families were asked to continue to complete pain diaries until they received the intervention, the number of actual days of completed diaries varied among subjects, ranging from 14 to 43 days. Therefore, the number of days with pain and number of days with missed activities were standardized to 30 days, allowing the presentation of number of days during the "previous month" at baseline.

## Results

**Figure 1:**
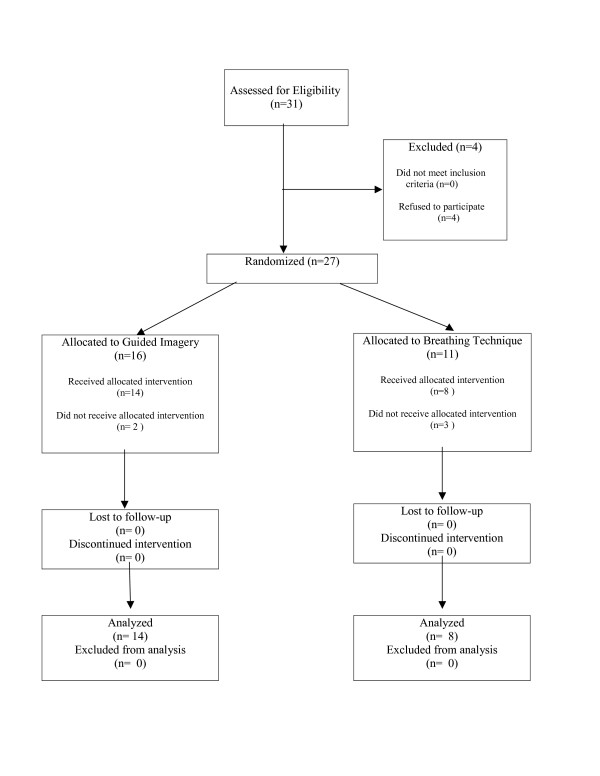
Flow diagram of study participants.

Between July 2000 and June 2002, 31 children were assessed for eligibility. All met inclusion criteria, however 4 refused to participate. [Figure 1] Twenty-seven children enrolled in the study and were randomized to receive either the breathing techniques or guided imagery. Three participants allocated to the breathing group and two allocated to the guided imagery group did not complete the baseline forms to start the interventions. Twenty-two received the intended treatments and all 22 completed the study.

Baseline characteristics of the final study population are summarized in Table 1. The two groups were similar in their age, gender, and psychological profiles. As noted in the table, children receiving guided imagery had significantly more days with pain during the baseline period than those learning breathing techniques alone (23 vs. 14.4 days, P = 0.04).

**Table 1:** Baseline Characteristics of Children and Parents by Intervention Group

	**Guided Imagery **(n = 14)	**Breathing **(n = 8)	**P-value**^1^
Child Factors			
% Male	23	50	0.17
Years of age	11.1	11.0	0.94
Months of pain at enrollment^2^	24.5	13.6	0.30
Days of pain previous month	23.0	14.4	0.04
Mean intensity of pain episodes	2.7	2.7	0.88
Days with a missed activity in previous month	4.0	1.3	0.12
Depression score^2^	6.6	7.1	0.80
Somatization score	6.5	4.0	0.15
Children's Somatization Inventory-total score	22.0	15.3	0.30
Anxiety score^2^	9.7	8.3	0.36
Perfectionism score^2^	9.0	8.1	0.43
Separation anxiety score	12.1	8.0	0.08
Temperament			
Anger	2.4	2.3	0.89
Distress	2.1	1.8	0.41
Fearfulness	2.2	2.4	0.54
Activity	2.3	2.8	0.24
Sociability	3.2	3.3	0.86
			
Parent Factors			
Ethnicity			
% Both parents Anglo	54%	50%	1.00
Anxiety score^2^	3.7	3.1	0.73
Somatization score^2^	6.9	3.8	0.13
Depression score^2^	6.8	6.3	0.87
			
Parenting Style			
Father caring score	4.1	3.6	0.59
Father overprotection score	3.5	4.4	0.26
Mother caring score	5.0	3.8	0.09
Mother overprotection score	4.3	4.0	0.57

^1 ^Chi-square test or Student t-test

^2 ^Geometric mean of natural logarithm transformed data

Table 2 summarizes the number of days of abdominal pain, mean intensity of pain episodes, and days of missed activities for both groups before, during, and after intervention. Compared to children learning breathing exercises alone, those learning guided imagery had a significantly greater decrease in days with pain during the initial month (67% vs. 21%, P = 0.05) and 2 months of follow up (82% vs. 45%, P < 0.01). In addition, those learning guided imagery had a significantly greater decrease in days with missed activities than children learning breathing exercises alone during the first month (85% vs. 15%, P = 0.02) and in the second month of follow up (95% vs. 77%, P = 0.05). There was no significant difference in the mean intensity of pain between the two treatment groups after one month as compared to baseline (52% vs. 41%, P = .58). Since these findings could be partially due to regression to the mean, we examined the correlation between baseline measures and the percent of improvement. The baseline days with pain was not significantly correlated with improvement at month 1 (r = 0.43, P = 0.08) or month 2 (r = 0.46, P = 0.07). In addition, there was no significant correlation between days with missed activities at baseline and improvement at month 1 (r = -0.09, P = .75) or month 2 (r = 0.10, P = .35). No adverse effects or symptom substitution were reported in either the guided imagery or breathing technique groups.

**Table 2:** Summary of days of pain, mean pain intensity, and days of missed activities.

**Time**	**Guided Imagery (n = 14)**	**Breathing Alone (n = 8)**	**P-Value**^1^
**Days with Pain**^2^			

Baseline	23.0 (17.6, 28.3)	14.4 (7.7, 21.1)	
Month 1	7.5 (2.9, 12.2)	11.3 (4.3, 18.2)	
Improvement	67%	21%	0.05
Month 2	4.2 (0.9, 7.5)	7.9 (3.7, 12.0)	
Improvement	82%	45%	< 0.01
			
**Mean Intensity of Pain Episodes**			
Baseline	2.5 (1.9, 3.2)	2.7 (2.0, 3.5)	
Month 1	1.2 (0.9, 1.5)	1.6 (0.6, 2.5)	
Improvement	52%	41%	0.58
			
**Days with Missed Activities**^2^			
Baseline	4.0 (1.5, 6.5)	1.3 (0.2, 2.4)	
Month 1	0.6 (0, 1.3)	1.1 (0.2, 2.1)	
Improvement	85%	15%	0.02
Month 2	0.2 (0,0.5)	0.3 (0,0.7)	
Improvement	95%	77%	0.05

^1 ^Student's t-test

^2 ^Both days with pain and missed activities are given per month.

**Figure 2:**
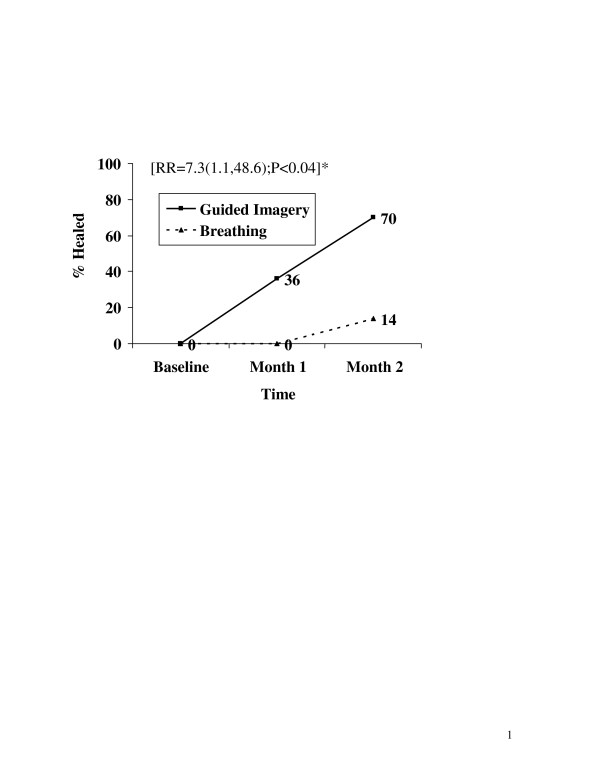
Outcome of percentage of subjects with ≤ 4 days of pain and no missed activities. (Healed) Relative Risk (RR), 95% confidence interval, and p-value were all calculated from the unadjusted generalized estimation equation model.

Figure 2 shows the percentage of participants who were "healed" (4 or less days of pain with no missed activities during each month) during the trial. During the 2 months of follow up the majority of children learning guided imagery and PMR were healed, while only a small percentage of those learning breathing exercises alone was healed (RR = 7.3, 95% C.I. [1.1, 48.6], P < 0.04). No child or parent factors confounded either of these results.

## Discussion

The primary finding from this study was that children suffering from RAP who learned guided imagery with progressive muscle relaxation were much more likely to improve and ultimately be healed than children learning breathing techniques alone. In addition, the greater therapeutic effect of guided imagery with PMR was sustained after termination of the intervention and occurred in spite of the children in this group who entered with more frequent complaints of abdominal pain.

Since Apley's description of recurrent abdominal pain in school-aged children in the 1950s, physicians and researchers have sought treatments for functional gastrointestinal disorders using the model most prevalent in the medical system–that of a biomechanical approach. Unfortunately, this approach has not led to effective therapies as evidenced by the recent evidence-based review of pharmaceutical therapies by the Cochrane Collaboration [10]. This review found just one effective pharmaceutical drug, not available in the United States, which was specific only for abdominal migraines which account for a small minority of patients with functional gastrointestinal disorders [51]. Recognizing the importance of an individual's psychological and social state, we have had to reassess our approach to this disorder to encompass a model much larger than what is currently being used–that of a biopsychosocial model. Although psychological interventions have been shown to be effective therapies for functional gastrointestinal disorders [20,21,52], pediatricians rarely consult psychologists for this group of patients [23].

As our knowledge of the brain-gut interaction has grown, researchers and physicians have utilized the mind-body techniques as effective therapy for functional gastrointestinal disorders. Gastrointestinal symptoms have improved with the use of hypnosis and relaxation training in adults with IBS [30,53]. In a case series of children with RAP, effectiveness was shown with a single session for training in self-hypnosis [29]. Prolonged improvement was demonstrated in another study using guided imagery in all subjects [36]. Our previous pilot study of guided imagery in children with RAP demonstrated significant therapeutic benefit with this intervention [37]. These mind-body techniques take advantage of one's own innate healing abilities thus empowering individuals to diminish their own pain, or other symptoms, through self-regulation.

In our protocol we specifically encouraged the participants to create their own image that represented their pain and a second image that would take their pain away. One young adolescent participant very vividly described a large, red, hot, immovable boulder to represent his pain. As his solution, he imagined first a trickle of rain, then a more forceful rain, and eventually, torrents of rain. The rain initially bounced off the hot rock, but then eventually cooled it–changing its color to a dull brown–broke the boulder into small pieces, and then washed it completely away. Most participants had no problem creating their own images at the first session and showed competence in doing this technique on their own.

There are four potential limitations of this study. First, all children did not have the same diagnostic evaluation, which might have differed between general pediatricians and pediatric gastroenterologists. However, we previously found no differences between children referred from generalists and specialists [39]. Our use of standard screening laboratory investigations, with additional testing tailored to the individual child is clinically recommended [54,55], and consistent with previously published studies [56]. Second, there is no evidence-based standard of care for this group of children, therefore we chose to control for the therapist's time and attention by teaching breathing exercises to our control group rather than use a wait list or other type of counseling setting. Third, it was impossible to blind the therapist to the treatment in this study. However, consistent with previously published methodologic standards [38] all other members of the research team were blinded to the treatment group. Both groups were referred to as "relaxation techniques" and the research assistant recording the outcomes was blinded to the treatment group. Our study protocol does not allow us to determine if the observed therapeutic response was due to progressive muscle relaxation, guided imagery, or both. However, guided imagery is generally considered a more powerful technique for pain syndromes and in clinical practice is most always preceded by progressive muscle relaxation [32,36,57]. Fourth, the decrease in days with pain might partly be due to regression to the mean, however this is not the case for missed activities or the primary outcome of being healed.

It is not surprising that a mind-body therapy, such as guided imagery, is effective for RAP as functional gastrointestinal disorders are theorized to be the result of a dysregulation of the brain-gut neuroenteric system, much like anovulatory bleeding is a dysregulation of the hypothalamic-pitutary-ovarian system [58]. Our study clearly shows that the response to guided imagery in this group of children with RAP was rapid, sustained, clinically effective and not associated with any apparent side effects. This intervention could easily be initiated at the first evaluation for abdominal pain then continued while completing any diagnostic work up. This would likely have benefits to the child whether or not they had an organic cause for their abdominal pain as it is an effective tool for coping with pain. Guided imagery could be presented to the patient and family as 'imagination therapy' and be done by the pediatrician, psychologist, social worker, child-life therapist, or nurse trained in guided imagery. By using this type of therapy early in the course of the evaluation and treatment of RAP it is possible, as in the adult studies [30,59], to reduce not only the number of days with pain with subsequent return to regular activities, but also reduce health care costs by decreasing the use of medical services.

## Conclusion

Guided imagery techniques along with progressive muscle relaxation is more effective than breathing and relaxation techniques for reducing pain episodes and missed activities in children with RAP. The response to guided imagery in this group of children with RAP was rapid, sustained, clinically effective and not associated with any apparent side effects.

This study is relevant to pediatrics as recurrent abdominal pain is a common complaint in children with very few treatments that have been found to be efficacious in the recent systematic reviews. Guided imagery is one type of self-regulation technique that has been beneficial in treating other pain syndromes and now found, in this study, to be beneficial in treating recurrent abdominal pain. Use of this type of therapy early in the course of the evaluation and treatment of RAP may reduce not only the number of days with pain with subsequent return to regular activities, but also reduce health care costs by decreasing the use of medical services.

## Competing interests

The author(s) declare that they have no competing interests.

## Authors' contributions

JW participated in study coordination and execution, and drafting of the manuscript

DS conceived the study and participated in its design and coordination

SA conceived the study and participated in its design and coordination

CM participated in study design, coordination, and execution

AC participated in study design, coordination, and execution

TB participated in study design, performed statistical analysis, and drafting of the manuscript

All authors read and approved the final manuscript.

## Pre-publication history

The pre-publication history for this paper can be accessed here:


